# A Comparative Proteomics Study of Autopsy and Fresh-Frozen Coronary Artery Samples

**DOI:** 10.1021/acs.jproteome.5c00152

**Published:** 2025-06-23

**Authors:** Xiaoke Yin PhD, Alicia Beele MSc, Konstantinos Theofilatos PhD, Ferheen Baig PhD, Maria Hasman PhD, Lukas E. Schmidt MSc, Joseph J. Boyle PhD, Adam W. Turner PhD, Clint L. Miller PhD, Gerard Pasterkamp MD, Stefan Stojkovic MD PhD, Johann Wojta PhD, Michael Joner MD, Manuel Mayr MD PhD

**Affiliations:** † National Heart and Lung Institute, 4615 Imperial College London, London, W12 0BZ, U.K.; ‡ Department of Cardiovascular Diseases, German Heart Centre Munich, TUM University Hospital, 80636 Munich, Germany; § School of Cardiovascular and Metabolic Medicine & Sciences, King’s College London, London, SE5 9NU, U.K.; ∥ Department of Internal Medicine II, Medical University of Vienna, 1090 Vienna, Austria; ⊥ Department of Genome Sciences, University of Virginia, Charlottesville, Virginia 22903, United States; # Division Laboratories and Pharmacy, 8124 University Medical Center Utrecht, 3584 CX Utrecht, Netherlands; ▼ DZHK (German Center for Cardiovascular Research), Partner Site Munich Heart Alliance, 80636 Munich, Germany

**Keywords:** Post-mortem interval, tissue proteomics, cardiovascular disease, protein degradation

## Abstract

Proteomic analyses of human tissues are often conducted on autopsy samples. However, no detailed comparative analysis between proteomic changes derived from autopsy samples and fresh-frozen samples has been undertaken. In this study, human left anterior descending (LAD) coronary artery samples (n = 94) from deceased patients were analyzed using nanoflow LC-MS/MS. Among consistently quantified proteins, 37% of the protein abundances exhibited significant correlations with the post-mortem interval (PMI), most of which are inverse. Notably, smooth muscle cell markers displayed substantial reduction with prolonged PMI. Conversely, positive correlations were observed for immunoglobulins, coagulation factors, and complement factors, including coagulation factor XII, plasminogen, and lactotransferrin. Comparative analyses of sex-specific protein changes in autopsy LAD samples versus fresh-frozen LAD samples (n = 65) showed no concordance. However, a robust correlation was observed between 2 different cohorts of fresh-frozen carotid endarterectomies (n = 120 and n = 200). This study represents the first large-scale proteomics investigation into the influence of PMI on the protein composition of the human vasculature, showing significant correlations with PMI for 37% of the quantified proteins. Our findings underscore potential discrepancies in the quantitative accuracy of proteomics data derived from autopsy samples. Consequently, results obtained from post-mortem specimens may not be reproducible in fresh-frozen samples.

## Graphical abstract

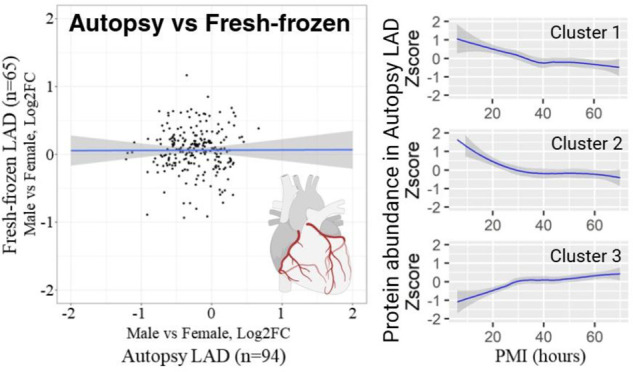

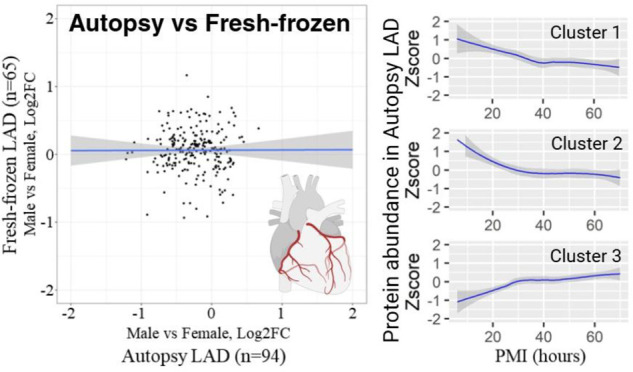

## Introduction

Despite advancements made by initiatives such as the Human Protein Atlas project, the comprehensive proteomic mapping of blood vessels remains inadequately addressed. Fresh-frozen human vessels, especially coronary arteries, are difficult to obtain. Protein identifications can be achieved through mass spectrometry (MS) from autopsy samples, as demonstrated in large-scale proteomic analyses of human coronary arteries,^1,2^ myocardium^3^ and other organs.^4^

The recently announced Proteomic Navigator of the Human Body (π-HuB) project will primarily utilize autopsy samples.^5^ A critical question arises whether the quantitative protein changes identified in autopsy samples can be reproduced in fresh-frozen samples. To our knowledge, no detailed comparative proteomic analyses between autopsy and fresh-frozen arteries have been conducted. Similarly, an assessment of the influence of the post-mortem interval (PMI) on protein quantification in vascular tissues had not yet been explored.

## Material and Methods

In the current study, left anterior descending (LAD) coronary artery samples (n = 94) were obtained from proximal and distal regions during autopsy at the German Heart Centre Munich with the PMI recorded. The study protocol was reviewed and approved by the ethics committee of the Technical University of Munich (reference number 325/18S). Written informed consent was provided by family members of deceased patients.

Each sample was mechanically homogenized using Lysing matrix D on a FastPrep homogenizer (MP Biomedicals) in guanidine buffer (4 M GuHCl, 50 mM sodium acetate, pH = 5.8, 25 mM EDTA, supplemented with Protease Inhibitor). Proteins were precipitated using 100% ethanol, resuspended in deglycosylation buffer (0.2 M Tris, 0.2 M sodium acetate, 0.1 M EDTA, 50 mM sodium phosphate, pH = 6.8), and deglycosylated using the following enzymes: endo-α-N-acetylgalactosaminidase, β1,4-galactosidase, β-N-acetylglucosaminidase, α-2–3,6,8,9-Neuraminidase (Glycoprotein Deglycosylation Kit, Merck Millipore 362280), Chondroitinase ABC (Sigma-Aldrich, C3667), Heparinase II (Sigma-Aldrich, H6512), and Endo-β1,4-galactosidase (Sigma-Aldrich, G6920). Samples were incubated for 1 h at 25 °C, followed by 24 h at 37 °C in agitation then dried using SpeedVac (Thermo Fisher Scientific). Subsequently, samples were reconstituted in ^18^O-water containing N-Glycosidase F (Glycoprotein Deglycosylation Kit, Merck Millipore) and incubated at 37 °C with agitation for 24 h. Afterward the samples were denatured in 6 M urea/2 M thiourea, reduced with 10 mM DTT at 37 °C for 1 h, and alkylated with 50 mM iodoacetamide at room temperature for 1 h in the dark. Proteins were precipitated by prechilled acetone overnight in −20 °C, resuspended in 0.1 M triethylammonium bicarbonate (TEAB, pH = 8.5, Sigma-Aldrich) and digested with Trypsin/LysC (Promega, enzyme:protein = 1:25) at 37 °C overnight. The digestion was stopped by using 1% trifluoroacetic acid (TFA). Peptides were purified using C_18_ cartridges on Bravo AssayMAP robot (Agilent). The cleaned peptides were SpeedVac dried, resuspended in 2% acetonitrile (ACN) containing 0.05% TFA and separated by nanoflow HPLC (U3000 RSLCnano, EASY-Spray C_18_ column, 50 cm x 75 μm, Thermo Fisher Scientific) using a trap-and-elute setup with a PepMap C_18_ 5 mm x 300 μm Trap Cartridge. The following gradient was used at 0.25 μL/min: 0–1 min, 1% B; 1–6 min, 1–6% B; 6–40 min, 6–18% B; 40–70 min, 18–35% B; 70–80 min, 35–45% B; 80–81 min, 45–99% B; 81–89.8 min, 99% B; 89.8–90 min 99–1% B; 90–120 min, 1% B; where A = 0.1% formic acid in H_2_O, B = 80% ACN, 0.1% formic acid in H_2_O. MS^1^ spectra were acquired on an Orbitrap Q Exactive HF mass spectrometer (Thermo Fisher Scientific) using full MS mode (resolution of 60,000 at 200 *m*/*z*) over the mass-to-charge (*m*/*z*) range 380–1500. Data-dependent MS^2^ scan (resolution of 15,000 at 200 *m*/*z*) was performed using higher-energy collisional dissociation (HCD) fragmentation on the top 15 ions in each full MS scan with dynamic exclusion enabled.

Proteome Discoverer (Thermo Fisher Scientific, version 2.4.1.15) was used to search RAW data against a human protein database (UniProtKB/Swiss-Prot version 2022_01, 20,376 protein entries) using MASCOT algorithm (version 2.6.0, Matrix Science). The mass tolerance was set at 10 ppm for precursor ions and 20 mmu for fragment ions. Trypsin was used as the digestion enzyme with up to two missed cleavages being allowed. Carbamidomethylation of cysteine was chosen as a fixed modification; oxidation of methionine, proline and lysine and deglycosylation of asparagine in the presence of ^18^O-water were chosen as variable modifications. The precursor signal intensity was used as quantitative value. Proteins identified with combined FDR < 0.01 and at least 2 unique peptides were exported and further statistic and bioinformatic analyses were carried out in the same way as previously described.^6^

Exported raw protein abundance values were filtered using a method to discriminate between random missing values and values that are consistently missing because of abundances below the limit of detection. Consistent missing values were identified and imputed with zeros when more than 90% missing values were observed in one region of the LAD and less than 10% in the other. Otherwise, proteins with >30% missing values were filtered out. All remaining missing values were imputed with the KNN method with k = 20.^7^ The relative quantities of the proteins were scaled using log2 transformation. The limma package was used for differential expression analysis (i.e., male vs female) using the eBayes algorithm and corrected for age.^8,9^ The P-values were adjusted for multiple testing using the Benjamini-Hochberg method. Spearman’s correlation was used for all association analyses.

Hierarchical clustering of protein abundance changes during the PMI was performed using proteins that showed significant correlations with PMI. The cluster abundance for each sample was calculated as the mean z-score of all proteins within that cluster. Trajectory analysis of the three largest clusters was performed using Generalized Additive Model. Nonlinear regression curves are shown with the 95% confidence intervals. Enrichment analysis was conducted using the Database for Annotation, Visualization, and Integrated Discovery (DAVID) tool.^10,11^ This analysis included pathway terms from Reactome, Kyoto Encyclopedia of Genes and Genomes (KEGG) and molecular function annotation from Gene Ontology.

The extracellular matrix protein differences between male and female were analyzed using the current data set (A) autopsy LAD samples (n = 94, 27.7% were from female), and three additional data sets: (B) fresh-frozen LAD samples (n = 65, 27.7% were from female) from control donor hearts and from patients undergoing cardiac transplantation (unpublished data); (C) fresh-frozen carotid endarterectomies (CEA) samples (n = 120, 26.7% were from female) from the Medical University of Vienna;^6^ and (D) fresh-frozen CEA samples (n = 200, 25.5% were from female) from the University Medical Center Utrecht (AtheroExpress cohort).^6^ Comparisons of protein fold changes between data sets (A) and (B) or between data sets (C) and (D) were performed to demonstrate the correlation of sex-specific protein changes after correcting for age.

## Results

The proteomics data were acquired by nanoflow LC-MS/MS with label-free quantitation. Protein abundances were normalized to the total protein abundance in each sample and showed consistent average log2 abundance across all 94 samples (CV = 0.96%). Consistently quantified proteins are listed in Table S1. The protein abundances in each sample were correlated to the post-mortem interval (PMI) of the sample and Spearman’s Rho values with adjusted P-values were calculated. Among these, 37% of the protein abundances exhibited significant correlations (adjusted P-value <0.05) with PMI, most of which were inverse (Figure 1a, Table S1). Unexpectedly, smooth muscle cell markers exhibited substantial reductions with prolonged PMI (*P* < 0.01, top left corner of the chart), including calponin-1 (CNN1), CNN2, CNN3, transgelin-2 (TAGLN2), and smoothelin (SMTN). Conversely, positive correlations with PMI (top right corner of the chart) were observed for immunoglobulins, coagulation factors, and complement factors, including plasminogen (PLG), lactotransferrin (LTF) and coagulation factor XII (F12). Two examples of protein abundance changes with increasing PMI were plotted: calponin-3 (CNN3), which decreased (Figure 1b), and coagulation factor XII (F12), which increased (Figure 1c). Linear regression (blue line) with 95% confidence intervals (gray shade area) is displayed in both plots.

**Figure 1:**
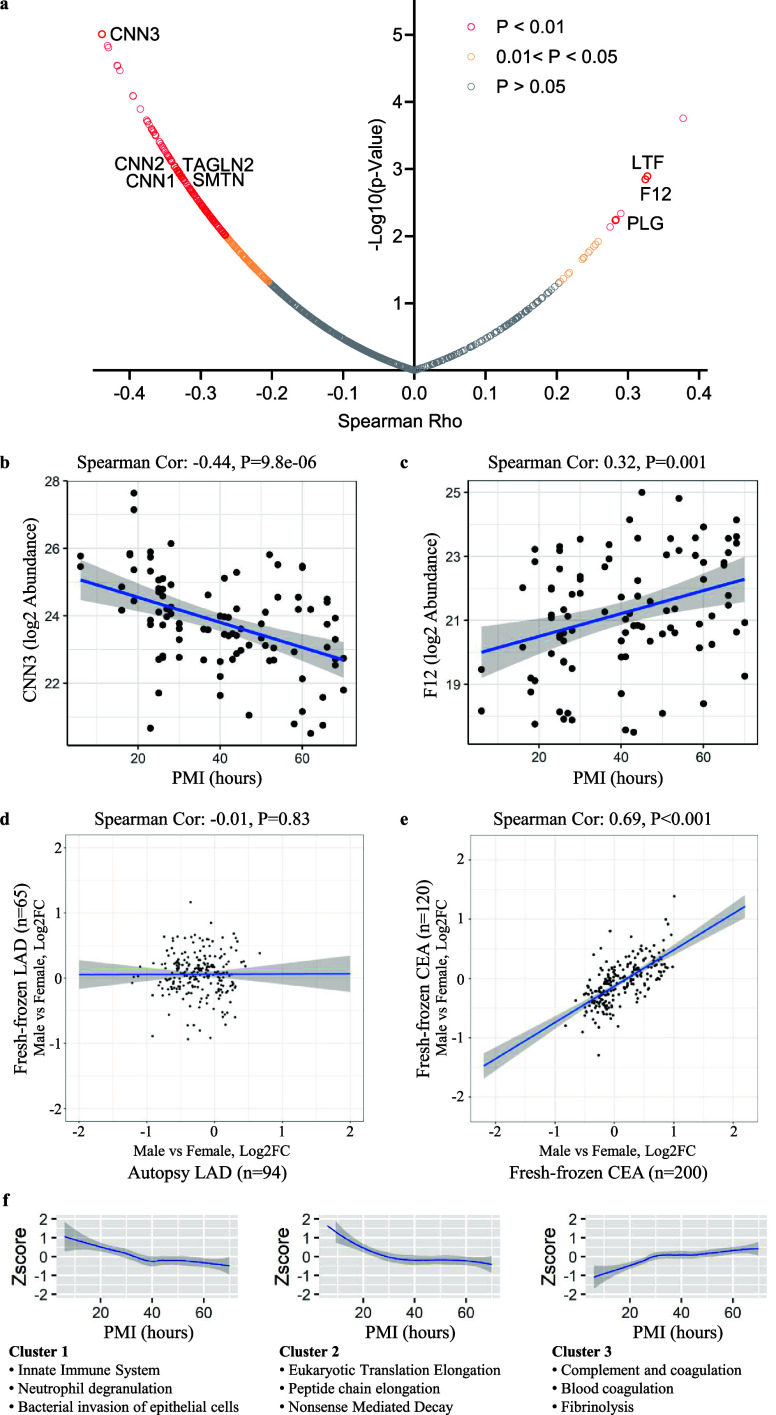
(a) The Spearman’s Rho correlation between protein abundances and post-mortem interval (PMI) in human left anterior descending (LAD) coronary artery autopsy samples (*n* = 94) is illustrated. The circles represent individual proteins. The *x*-axis displays the Spearman Rho value, and the *y*-axis shows the -log10­(p-value). Smooth muscle cell (SMC) markers exhibited a significant negative association (Spearman Rho <0, adjusted P-value <0.05) with PMI, while immunoglobulins, coagulation factors, and complement factors demonstrated a significant positive association (Spearman Rho >0, adjusted P-value <0.05). SMC markers: calponin-1 (CNN1), calponin-2 (CNN2), calponin-3 (CNN3), transgelin-2 (TAGLN2), and smoothelin (SMTN); coagulation factor XII (F12), lactotransferrin (LTF), and plasminogen (PLG). (b) Spearman’s correlation plot and linear regression line (blue) with 95% confident interval (gray area) showed negative association of CNN3 log2 abundance and PMI. (c) Positive association of PMI with the log2 abundance of F12. (d) In the proteomic comparison of sex-related extracellular protein changes using autopsy and fresh-frozen left anterior descending (LAD) coronary arteries, no correlation was observed between the two types of samples. Data were corrected for age differences. The gray areas represent the 95% confidence intervals of the linear regression line. (e) A strong correlation was evident when performing the same comparison between two independent data sets using fresh-frozen samples obtained from carotid endarterectomies (CEA). (f) Hierarchical clustering of protein abundance changes over PMI revealed distinct clusters, using proteins that showed significant correlations with PMI. Trajectory analysis of the three largest clusters was performed. Nonlinear regression curves are shown, with gray bands indicating the 95% confidence intervals. Based on enrichment analysis, the top three functional and pathway terms are provided for each cluster. The 3 clusters showed distinct trajectory curves and functional and pathway terms.

To assess the concordance between autopsy and fresh-frozen samples, we conducted a male versus female comparison using abundances of extracellular matrix (ECM) and ECM-associated proteins, which are expected to be less affected by post-mortem degradation compared to cellular proteins. Surprisingly, comparative analyses of sex differences between autopsy LAD samples (data set A) and fresh-frozen LAD samples (data set B) revealed no concordance in protein quantification with Spearman correlation Rho = −0.01 and P = 0.83 (Figure 1d). These findings indicate that the protein changes observed between males and females in the proteomic analysis of autopsy LAD samples could not be reproduced using fresh-frozen LAD samples. In contrast, a strong correlation was observed in a sex-based comparison conducted on two independent cohorts of fresh-frozen carotid endarterectomy (CEA) samples (data set C vs data set D, Spearman correlation Rho = 0.69, *P* < 0.001) (Figure 1e). This demonstrates that sex-related proteomic differences are reproducible when using fresh-frozen samples, but not when using autopsy-derived tissue.

When we examined protein abundance variations across samples, distinct clusters emerged (Figure 1f). Cluster 1, which was primarily enriched for proteins in the innate immune system, neutrophil degradation, and bacterial invasion of epithelial cells, displayed a gradual decrease with increasing PMI. Cluster 2, containing proteins related to eukaryotic translation elongation, peptide chain elongation and nonsense mediated decay, exhibited a rapid decline in the first 30 h after death, followed by a slower decrease. In contrast, cluster 3—a small number group of proteins involved in complement and coagulation and fibrinolysis—showed a gradual “increase” in protein abundance over time after death. These findings highlight that not all proteins degrade at the same rate. Some proteins appeared to “increase in abundance” when we normalized the protein raw abundance values to the total protein abundance of the sample, which can lead to misinterpretation of quantitative comparisons.

## Discussion

Our findings suggest that a similar number of protein identifications alone is not sufficient to conclude that autopsy samples can be reliably used for quantitation. Within the first 30 h post-mortem, many protein abundances changed 2- to 4-fold in a linear manner, exceeding the typical fold change observed in previous comparisons. While earlier sampling generally results in less protein degradation,^12^ any degree of protein degradation poses a challenge for normalization in quantitative proteomics. Notably, degradation begins within hours after death and progresses continuously, eventually affecting the majority of proteins. We did not observe a definitive PMI threshold within which sample quality could be assured. Proteins that degrade more slowly may appear to “increase” in abundance when standard workflows normalize against the total protein content. Therefore, quantitative proteomics data from post-mortem samples may be unreliable, even when collected at the same PMI.

Due to their proximity to blood, vessels are particularly susceptible to immune cell infiltration and extravasation of plasma proteins, which can influence their molecular composition. Notably, there was also a significant impact on SMCs. The cessation of blood flow halts oxygen supply, causing a rapid shift from aerobic toward anaerobic respiration.^13^ This shift triggers enzymatic proteolysis, leading to decomposition of myofilaments linked to the rigor mortis process, ususally 24–48 h post-mortem.^14^ While this process is well-described in skeletal muscle, it may also occur in vascular SMCs. Different muscle types exhibit distinct degradation patterns,^15^ and cardiac troponin has been utilized to estimate the PMI for up to 96 h.^16^

While protein identification is of course feasible from autopsy samples, their accuracy for quantifying protein levels does not match that of fresh-frozen samples. This limitation is evidenced by the weak correlation in log2 fold changes observed between male and female comparisons of LAD autopsy samples versus fresh-frozen LAD samples. In contrast, sex-based comparisons between two independent cohorts of fresh-frozen CEA samples showed strong correlations. These findings imply that post-mortem protein degradation is not uniform, and preservation of protein levels in autopsy samples is less reliable than in fresh-frozen tissues.

Previous proteomic studies using autopsy material reported a substantial reduction—up to 60%—in tricarboxylic acid (TCA) cycle proteins in fibrous plaque-enriched samples compared to normal intima.^1^ However, our study reveals that many mitochondrial protein abundances are already significantly reduced with increasing PMI (Table S1), including ATP synthase subunits (ATP5F1C, ATP5PO), TCA cycle proteins (SDHB), and components of oxidative phosphorylation (COX4I1, NDUFA5). Another proteomic study comparing coronary and large arteries, conducted using samples collected within 72 h post-mortem, reported enrichment of collagens and integrins (e.g., COL15A1 and ITGB4) in coronary arteries.^17^ We have observed a marginally significant decrease in COL15A1 and ITGB1 (p = 0.076 and 0.090, respectively), and a significant reduction in COL10A1 as PMI increased. Furthermore, fibulin-5, a protein involved in elastin assembly and microfibril interaction, was previously reported to be over 6-fold more abundant in large arteries compared to other heart regions.^17^ Yet, in our study, fibulin-5 levels increased significantly with PMI (p = 0.006). These reported protein changes may arise from varying protein degradation rates across functional groups and tissue types, casting doubt on the reliability of prior findings utilizing autopsy samples without assessing the effects of PMI. Similarly, tissue-specific, gene-specific, and genotype-dependent RNA degradation is also observed in post-mortem samples and is PMI-dependent.^18^ In a recent study,^19^ consistent with our proteomic data, only RNA extracted from FFPE sections of human coronary arteries from fresh explanted hearts yielded reliable sequencing results, whereas RNA from autopsy samples (with post-mortem intervals ranging from 12 to 72 h) did not.

This study is the first large-scale proteomics investigation into the impact of PMI on the protein composition of human blood vessels. Significant correlations with PMI were observed for nearly 40% of consistently quantified proteins, underscoring potential discrepancies in the quantitative accuracy of proteomics data from autopsy samples. Additional validation of putative protein changes identified in autopsy samples with fresh tissue is essential. Although antibody-based methods were not evaluated in this study, they could be similarly affected, depending on the susceptibility of specific epitopes to degradation. In the omics era, autopsy samples may compromise the quantitative accuracy of molecular profiling, underscoring the importance of biobanking fresh tissues.

## Conclusion

In summary, while protein identifications are feasible from autopsy samples, post-mortem protein degradation is not uniform, making protein levels in autopsy samples less reliable than in fresh-frozen samples. Consequently, quantitative findings obtained from post-mortem specimens should be validated in fresh-frozen samples. Given the heightened susceptibility of RNA to degradation, these concerns extend to transcriptomics, including resources like the Genotype-Tissue Expression project.

## Supplementary Material





## Data Availability

The data sets generated and analyzed during the current study have been deposited to the ProteomeXchange Consortium via the PRIDE partner repository under the data set identifier PXD052071.

## Notes

The Spearman’s correlation between protein abundances and post-mortem interval (PMI) in human left anterior descending (LAD) coronary artery autopsy samples (*n* = 94) (XLSX)
Description of Supporting Information file content (PDF)

## Abbreviations

CEA: carotid endarterectomy
CNN: calponin
ECM: extracellular matrix
F12: coagulation factor XII
LAD: left anterior descending
LTF: lactotransferrin
PLG: plasminogen
PMI: post mortem interval
SMC: smooth muscle cell
SMTN: smoothelin
TAGLN: transgelin
