# Supplementary material for: A Comparative Proteomics Study of Autopsy and Fresh-Frozen Coronary Artery Samples
Source: J Proteome Res. 2025 Jun 23;24(7):3154–9. doi: 10.1021/acs.jproteome.5c00152 (PMC12235707; doi:10.1021/acs.jproteome.5c00152)
Supplement: Supplementary file 2 [file pr5c00152_si_002.pdf]

SUPPORTING INFORMATION

A Comparative Proteomics Study of Autopsy and Fresh-Frozen Coronary Artery Samples

Xiaoke Yin, PhD[1], Alicia Beele, MSc[2], Konstantinos Theofilatos, PhD[3], Ferheen Baig, PhD[3], Maria Hasman, PhD[3], Lukas E. Schmidt, MSc[4], Joseph J Boyle, PhD[1], Adam W. Turner, PhD[5], Clint L. Miller, PhD[5], Gerard Pasterkamp, MD[6], Stefan Stojkovic, MD, PhD[4], Johann Wojta, PhD[4], Michael Joner, MD[2], Manuel Mayr, MD, PhD[1,4*]

[1] National Heart and Lung Institute, Imperial College London, London, W12 0BZ UK

[2] Department of Cardiovascular Diseases, German Heart Centre Munich, TUM University Hospital, 80636 Munich, Germany

[3] School of Cardiovascular and Metabolic Medicine & Sciences, King's College London, London, SE5 9NU, UK

[4] Department of Internal Medicine II, Medical University of Vienna, 1090 Vienna, Austria

[5] Department of Genome Sciences, University of Virginia, Charlottesville, VA 22903, USA

[6] Division Laboratories and Pharmacy, University Medical Center Utrecht, 3584 CX Utrecht, Netherlands

*** Corresponding Author**

Professor Manuel Mayr - *MD, PhD, National Heart and Lung Institute, Imperial College London,86 Wood Lane, London, W12 0BZ, UK*; Email: m.mayr@imperial.ac.uk

The following supporting information is available free of charge at ACS website http://pubs.acs.org

- Table S1. The Spearman's correlation between protein abundances and post-mortem interval (PMI) in human left anterior descending (LAD) coronary artery autopsy samples (n=94).

- File name: Table S1 Protein Abundance to PMI correlation.xlsx
- File type extension: Microsoft Excel xlsx file
